# Beta-Cell ARNT Is Required for Normal Glucose Tolerance in Murine Pregnancy

**DOI:** 10.1371/journal.pone.0077419

**Published:** 2013-10-24

**Authors:** Sue Mei Lau, Kuan Minn Cha, Ayesha Karunatillake, Rebecca A. Stokes, Kim Cheng, Mark McLean, N. W. Cheung, Frank J. Gonzalez, Jenny E. Gunton

**Affiliations:** 1 Diabetes and Transcription Factors Group, Garvan Institute of Medical Research, Sydney, Australia; 2 Prince of Wales Clinical School, Faculty of Medicine, University of New South Wales, Sydney, Australia; 3 Department of Diabetes and Endocrinology, Westmead Hospital, Sydney, Australia; 4 Faculty of Medicine, University of Western Sydney, Sydney, Australia; 5 Faculty of Medicine, University of Sydney, Sydney, Australia; 6 Laboratory of Metabolism, National Cancer Institute, Bethesda, Maryland, United States of America; 7 St Vincent’s Clinical School, University of New South Wales, Sydney, Australia; Monash University, Australia

## Abstract

**Aims:**

Insulin secretion increases in normal pregnancy to meet increasing demands. Inability to increase beta-cell function results in gestational diabetes mellitus (GDM). We have previously shown that the expression of the transcription factor *ARNT* (Aryl-hydrocarbon Receptor Nuclear Translocator) is reduced in the islets of humans with type 2 diabetes. Mice with a beta-cell specific deletion of *ARNT* (β-ARNT mice) have impaired glucose tolerance secondary to defective insulin secretion. We hypothesised that ARNT is required to increase beta-cell function during pregnancy, and that β-ARNT mice would be unable to compensate for the beta-cell stress of pregnancy. The aims of this study were to investigate the mechanisms of ARNT regulation of beta-cell function and glucose tolerance in pregnancy.

**Methods:**

β-ARNT females were mated with floxed control (FC) males and FC females with β-ARNT males.

**Results:**

During pregnancy, β-ARNT mice had a marked deterioration in glucose tolerance secondary to defective insulin secretion. There was impaired beta-cell proliferation in late pregnancy, associated with decreased protein and mRNA levels of the islet cell-cycle regulator cyclinD2. There was also reduced expression of *Irs2* and *G6PI*. In contrast, in control mice, pregnancy was associated with a 2.1-fold increase in ARNT protein and a 1.6-fold increase in cyclinD2 protein, and with increased beta-cell proliferation.

**Conclusions:**

Islet ARNT increases in normal murine pregnancy and beta-cell ARNT is required for cyclinD2 induction and increased beta-cell proliferation in pregnancy.

## Introduction

Gestational diabetes (GDM) and diabetes in pregnancy (DIP) together affect 5–10% of women in Western countries, and prevalence is increasing worldwide. The recent consensus statement recommending revision of the diagnostic criteria would further increase the number of women diagnosed with GDM [1], [2]. GDM is defined as glucose intolerance first diagnosed during pregnancy, whereas DIP also includes all women with glucose intolerance in pregnancy, regardless of time of diagnosis [3].

In normal pregnancy, there is a progressive increase in insulin resistance from mid-gestation through to late gestation. To maintain normal glucose tolerance, beta-cell function is upregulated through increased beta-cell size (hypertrophy), increased beta-cell number (proliferation), and improved beta-cell function (insulin secretion) [4], [5]. Inability to increase beta-cell function in order to match the metabolic demands of pregnancy results in GDM [6], [7].

Autopsy studies in pregnant women have demonstrated that islet area as a proportion of non endocrine pancreas increases in pregnancy. One study reported a 2.1-fold increase in islet mass in pregnancy, with a 12.6% increase in the proportion of beta-cells per islet, indicative of hyperplasia [8]. Another reported greater numbers of small islets, with a 1.5-fold increase in beta-cell area [9]. Mean 24 hour insulin concentrations are 50% higher in the third trimester compared with the non-pregnant state [6]. In rats, beta-cell mass is increased 2.5 fold at the end of gestation [10] due to increased size and number of beta-cells. Beta-cell proliferation was assessed with 5-bromo-2-deoxyuridine (BrdU) staining. It peaked at a ∼17 fold increase during rodent pregnancy [11], [12].

How beta-cell function is upregulated in pregnancy is not fully understood. In humans, prolactin and human placental lactogen (PL) levels increase and may drive an increase in beta-cell mass or function [12]. In rodents, gestational augmentation of beta-cell mass is controlled by the placental lactogens PLI and PLII [13] and growth hormone which activates the rodent, but not human prolactin receptor [14]. These hormones signal through a number of pathways, of which Jak-STAT5 is thought to be the most important [15]–[17]. However, less is known about the role of other beta-cell transcription factors in pregnancy. As women with GDM are at much greater risk of developing type 2 diabetes in later life, it is possible that the beta-cell dysfunction associated with both these conditions may originate from the same cellular defects.

We previously reported *ARNT* (Aryl-hydrocarbon Receptor Nuclear Translocator, also called hypoxia inducible factor 1β (HIF-1β)) to be a susceptibility factor for beta-cell dysfunction and type 2 diabetes (T2D) [18]. *ARNT* mRNA was reduced by 90% in human T2D islets (p<0.001). In that study, 3 of the 5 female islet donors with T2D had a known personal history of GDM, versus 0 of 5 women with normal glucose tolerance, suggesting that decreased *ARNT* may also be relevant to human GDM [18]. Eight (8) week old female mice with a beta-cell specific knockout of *ARNT* (β-ARNT mice) had mildly impaired glucose tolerance, with preservation of fasting glucose [18], [19]. This was associated with a loss of first phase glucose stimulated insulin secretion. Body weight and insulin sensitivity were unchanged. β-ARNT females had significant deterioration of glucose tolerance during pregnancy, which was not seen in controls [18].

The aim of this study was to investigate the mechanisms of ARNT regulation of beta-cell function and glucose tolerance in pregnancy.

## Materials and Methods

### β-ARNT Mice

The β-ARNT (beta-cell specific Aryl-hydrocarbon Receptor Nuclear Translocator knockout) mice were generated as previously reported [18], [20], by breeding transgenic mice expressing *Cre*-recombinase under control of the rat insulin promoter (RIP-*Cre*) and homozygous *Arnt* floxed mice. One copy of RIP-*Cre* produces the phenotype, thus; *Arnt* fl/fl, RIP-*Cre*(*+*) = β-*Arnt* mice, whereas *Arnt* fl/fl, RIP-*Cre*(−)  =  floxed-controls (FC). Our RIP-*Cre* colony has no differences in glucose tolerance testing compared to wild types or floxed-controls (p>0.6 for all time points at GTT).

Mice were inbred C57Bl/6 for more than 12 generations. All study mice were bred exclusively from FC females (not β-ARNT females) for >10 generations to exclude fetal programming effects. The mice were housed in our Biological Testing Facility with a standard 12 hour light/dark cycle and fed normal chow and water *ad libitum.*

### Ethics

All animal studies were approved by the Garvan Animal Ethics Committee and carried out in accordance with their guidelines and national legislation. Minimum required quantities of blood were collected for each experiment. AEC approvals were #06.04 and #10.35.

### Timed Mating and Gestational Care

**Figure 1:**
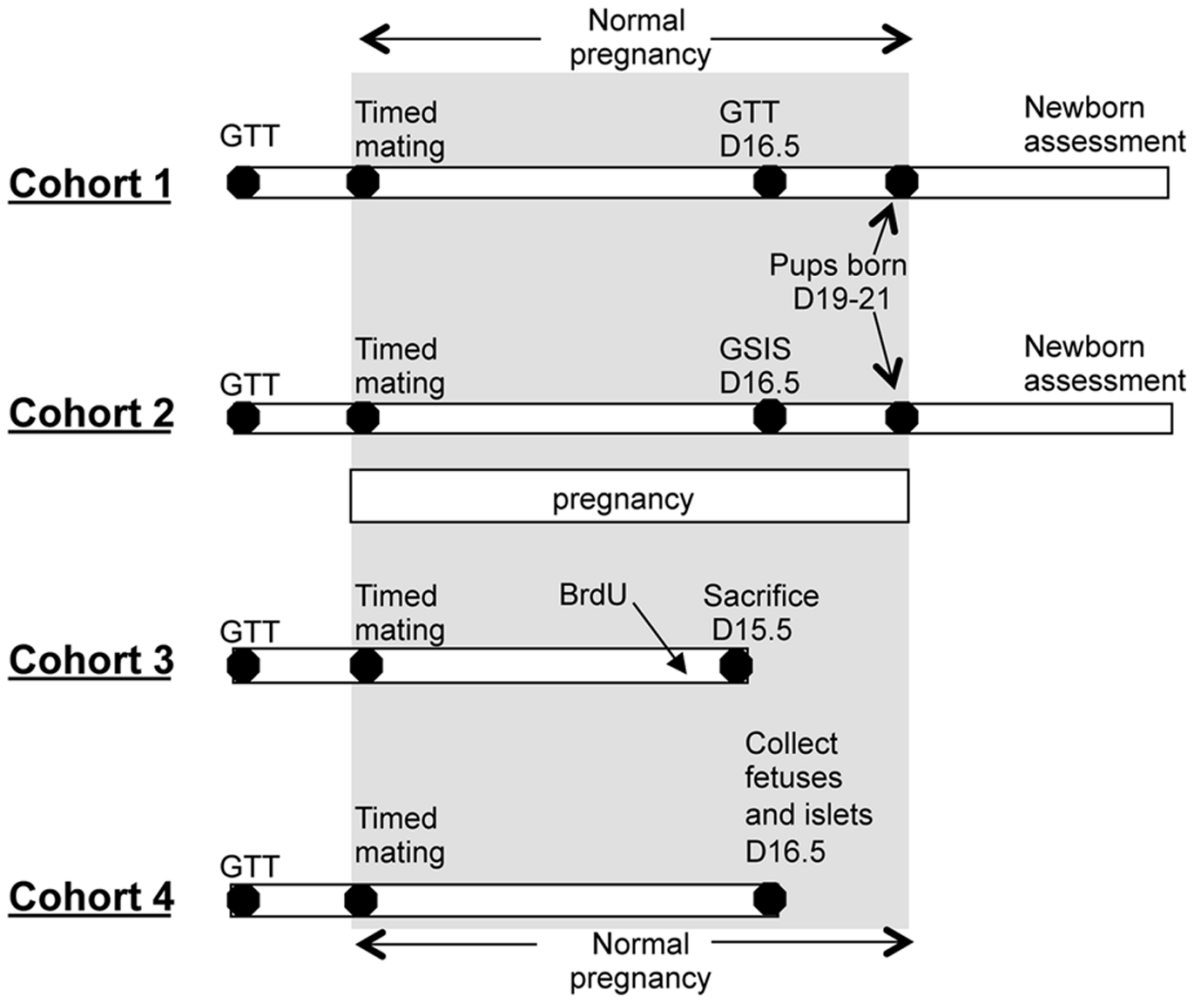
Timelines for the 4 cohorts of pregnant mice. GTT (glucose tolerance testing) was performed on all female mice prior to pregnancy. After timed mating, GTT was repeated on day 16.5 of gestation in cohort 1, and glucose-stimulated insulin secretion (GSIS) in cohort 2. Newborn pups in these 2 cohorts were weighed at day 1.5. Female offspring from cohort 1 were weighed weekly from weaning and timed mated at 10–12 weeks of age with a GTT at day 16.5. GTT and GSIS were performed prior to pregnancy. In cohort 3, BrdU was administered 16 hours before sacrifice at day 15.5 of gestation. In cohort 4, fetuses and islets were collected at day 16.5 of gestation.

Timelines for the cohorts are mice are shown in **Figure 1**. Twelve week old β-ARNT female mice were mated with FC males, and FC females were mated with β-ARNT males. Because the breeding plan has one parent being *Cre*+, in all pregnancies, ∼50% of offspring will be β-ARNT and ∼50% FC.

Glucose tolerance tests (GTT) were performed on females 2–3 weeks prior to pregnancy. Date of conception was determined by vaginal plugging the following morning, which was therefore day 0.5 of gestation (G0.5). Pregnant dams were singly housed from conception until delivery or sacrifice. Four cohorts were studied (Figure 1). Cohort 1 had glucose tolerance testing at day G16.5 of pregnancy, cohort 2 had *in vivo* measurement of glucose stimulated insulin secretion (GSIS) at day G16.5. Cohort 3 had pancreas collection on day G15.5, which is around the reported peak beta-cell proliferation in pregnancy [12]. These mice received intraperitoneal BrdU (60 mg/kg) 16 hours before pancreas collection. Cohort 4 had islets isolated at day G16.5 for gene expression or protein measurements.

Offspring from cohorts 1 and 2 were weighed at post-natal day 1.5 of age. Fetuses in group 4 were weighed and blood was collected for measurement of glucose and insulin.

### Metabolic Testing

Glucose tolerance tests were performed as previously reported [19], [21]. After an overnight fast, 20% dextrose was administered at a dose of 2 g/kg by intraperitoneal injection. Blood glucose was measured via glucometer (Freestyle Lite, Abbott, Australia) prior to, and at 15, 30, 60, 90 and 120 minutes after the dextrose injection.

Glucose stimulated insulin secretion (GSIS) was measured after an overnight fast. Intraperitoneal glucose was given at 2 g/kg and the test was otherwise performed as previously reported [18], [19]. 15–20 µL of blood was collected prior to, and at 2, 5 and 20 minutes after the dextrose injection. Blood was immediately centrifuged and the supernatant stored at−20°C for insulin ELISA assay. In both of the above procedures, blood was collected via a distal tail nick.

### DEXA Scanning

Dual energy X-ray absorptiometry (DEXA) scanning was performed using a Lunar PIXImus2 (GE Healthcare, Port Washington, NY, USA) as previously reported [22].

### Pancreas and Islet Collection

The pancreas was dissected, weighed and fixed in 10% formalin in PBS overnight at 4°C before embedding flat in paraffin blocks. Islets were isolated as previously reported [18], [21]. In brief, the bile duct was cannulated and collagenase (Liberase, Roche, USA, in M199 media) was injected. Each distended pancreas was digested for 18 minutes at 37°C, after which M199 and bovine calf serum (BCS) were added and tubes were alternately shaken and vortexed, then centrifuged. Islets were separated from exocrine tissue using a Ficol gradient. The islets were washed and centrifuged with M199+BCS and the supernatant was aspirated, leaving a pellet that was resuspended and placed on ice for 4 minutes. This last step was repeated and the islet pellet was snap frozen in liquid nitrogen and stored at−80°C.

### Assays

Plasma insulin was assayed using the Crystal Chem ELISA kit (Chicago USA) according to the manufacturer’s instructions. Triglycerides were assayed from fasting plasma using the colorimetric assay from Roche (Basel, Switzerland).

### Histology

#### Immunofluorescence: BrdU

Pancreatic sections were cut at 7 µm thickness. Three widely separated sections were chosen per pancreas. After dewaxing and rehydration, antigen retrieval was performed at 125°C for 1 minute in Dako Target Retrieval Solution pH 9.0 (Dako, Carpinteria, NJ). Sections were incubated with anti-BrdU antibody (BD Biosciences Franklin Lakes, NJ) and rabbit anti-insulin antibody (Cell Signaling, Danvers, MA) in blocking buffer. After 3 washes, sections were incubated with secondary antibodies (Cy2 anti-mouse and Cy3 anti-rabbit, Dako) in blocking buffer, with DAPI (Dako). After three further washes and a water-rinse, cover slips were mounted and slides were stored at 4°C until imaging (see below).

#### Immunofluorescence: cleaved caspase-3

Caspase 3 is cleaved when it is activated during the process of apoptosis. Cleaved caspase-3 immunofluorescence was performed as per [23]. Antigen retrieval was performed in a 10 mM sodium citrate buffer in a 90°C water-bath for 25 minutes. The primary antibodies were rabbit anti-cleaved caspase-3 antibody (R&D Systems Minneapolis, MN) and guinea pig polyclonal anti-insulin antibody (Dako). Secondary antibodies were anti-rabbit Cy3 (Dako) and anti-guinea pig Alexa Fluor 488 Ig (Invitrogen, Carlsbad, CA), with DAPI.

### Quantitation of BrdU Staining and Islet Area

Sections were viewed with a Zeiss inverted Axiovert 200 M confocal microscope (Carl Zeiss Thornwood, NY) using Axiovision software version 4.6.3 (Carl Zeiss). Positive cells were counted by 2 independent observers blinded to the mouse genotype. Pictures of all islets in each section were analysed for the number of BrdU-positive (+), insulin+ cells per unit of beta-cell area (insulin positive area). The BrdU labelling index was calculated using the formula:

BrdU labelling index  =  (number of BrdU + cells in all islets/total area of all islets ( µm^2^)) * 10 000, and was calculated for each section from each mouse.

### Pancreatic Section Area and Beta-cell Mass

After immunofluorescence analysis was completed, coverslips were removed. Routine H&E counterstaining was performed. The stained section was scanned and the total pancreatic section area calculated using NIH 1.63 software.

Beta-cell mass was calculated as previously reported [21], as follows: beta-cell mass  =  (total beta-cell area in 3 sections/total area of 3 sections) * pancreatic weight.

### Quantification of Cleaved Caspase-3 Staining

The procedure was similar to BrdU analysis, except that 1 section was viewed per mouse. Caspase staining index was calculated using the formula:

Caspase staining index  =  (total number of caspase + cells in all islets/total area of all islets ( µm^2^)) * 10 000.

### Gene Expression

After tissue homogenisation in RLT buffer (Buffer RLT Lysis Buffer, Qiagen Valencia, CA), RNA was extracted and purified from islets as previously reported [21]. cDNA was synthesised from 0.5 µg of RNA using random hexamer primers as previously reported and real time PCR was performed as previously reported [21]. PCR primers are available on request.

### Western Blotting

Proteins were separated by SDS-PAGE. After transfer onto PDVF membrane and blocking in 5% milk in TBST, membranes were incubated with anti-Cyclin D2 antibody (1∶200 dilution, Santa Cruz Biotechnology, Santa Cruz, CA) and donkey anti-rabbit secondary antibody (1∶1000, Thermo Fisher Scientific, Rockford, IL). Proteins were visualised by enhanced chemiluminescence (Western Blotting Luminol Reagent, Santa Cruz Biotechnology, Santa Cruz, CA), using a BioRad Molecular Imager. Alpha tubulin was used as a loading control. The antibody was from Abcam, Cambridge, UK. A goat anti-mouse secondary antibody (Thermo Fisher Scientific, Rockford, IL) was used. For the ARNT Western blots, Anti-HIF-1β/ARNT antibody was used to stain for (1∶1000 dilution, BD Biosciences, San Jose, CA). A goat anti-mouse secondary antibody was used. Bands were normalised to TBP (TATA-binding protein) housekeeping protein.

Band intensity for proteins and their loading controls were quantitated using ImageJ software.

### Chromatin Immunoprecipitation

ChIP was performed as previously reported [21], except that ARNT antibodies were used (BD Biosciences). CyclinD2 promoter primers are available on request.

### Statistics

Statistical analyses used t-tests for 2 groups, or ANOVA and post-hoc testing with Bonferroni correction for >2 groups unless otherwise stated, using SPSS version 20. Data are expressed as mean ± SEM unless otherwise specified. A p-value of <0.05 was considered significant.

## Results

### β-ARNT Mice had Normal Fertility

β-ARNT dams were timed mated as indicated in Figure 1. They had normal fertility and remained systemically well through pregnancy. Maternal body weights were similar at gestational day 16.5 (31.8±0.9 versus 30.4±1.2 g in floxed controls (FC), p = ns). Litter sizes were similar in β-ARNT and FC pregnancies (6.7±0.5 versus 6.3±0.4, p = ns). Rates of pregnancy loss or pup abandonment did not differ.

### Glucose Tolerance in Pregnancy

**Figure 2:**
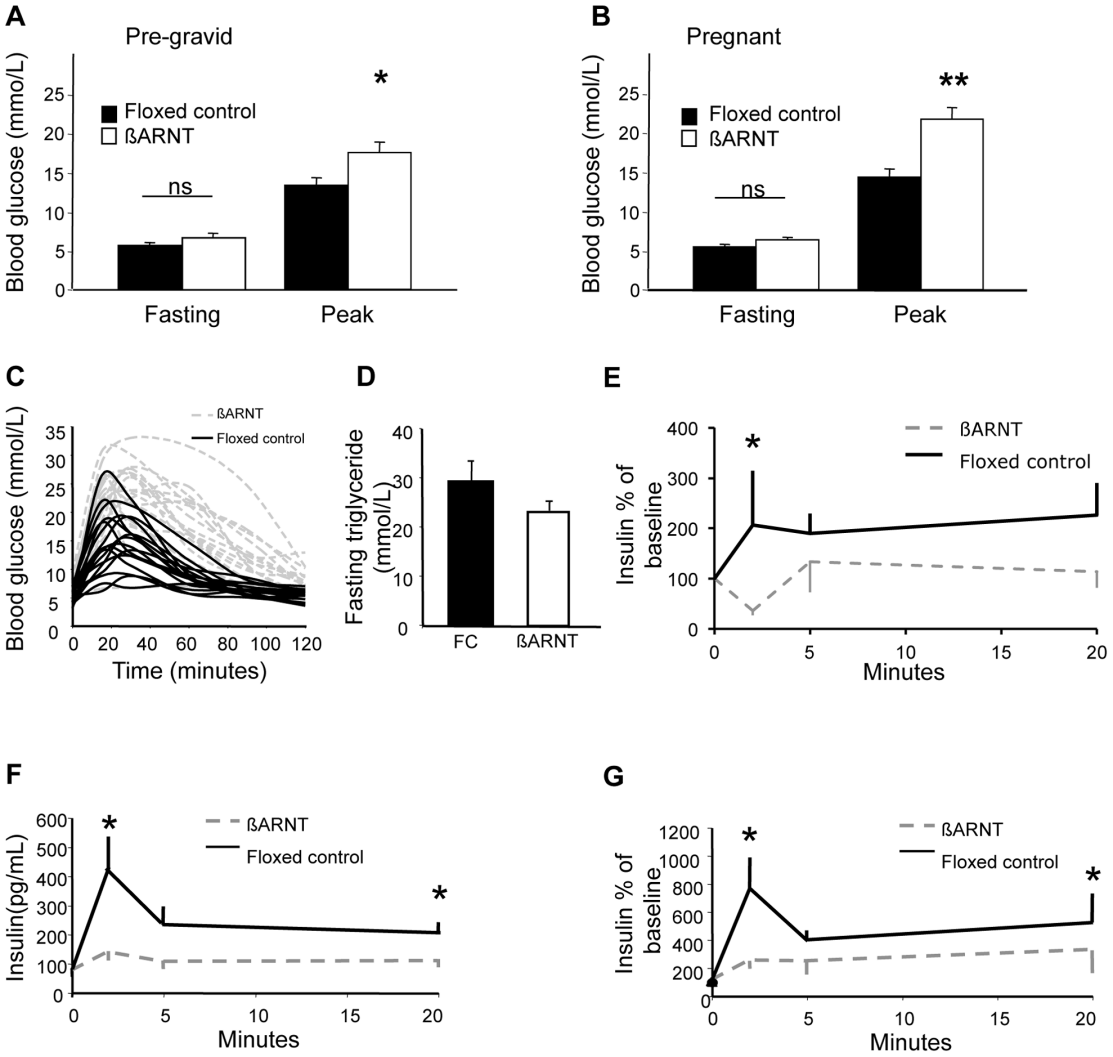
Glucose metabolism in ß-ARNT and floxed control females. (A) Pre-gravid fasting and peak blood glucose levels in ß-ARNT (n = 19) and floxed control (n = 19) females. *p<0.05, t-test. Bars represent mean±SEM. (B) Late gestational fasting and peak blood glucose levels in ß-ARNT (n = 19) and floxed control (n = 19) females. **p<0.01, t-test. Bars represent mean±SEM. (C) Glucose tolerance curves for ß-ARNT and floxed control pregnancies. Each curve represents an individual mouse. (D) Fasting triglycerides in pregnant ß-ARNT and floxed control (FC) females. Bars represent mean±SEM. (E) Decreased first phase insulin secretion in non pregnant ß-ARNT versus floxed control females. *p<0.05. Data points represent mean±SEM. (F) Decreased first and second phase insulin secretion in pregnant ß-ARNT (n = 12) versus floxed control (n = 14) females. Insulin expressed in absolute values. *p<0.05. Data points represent mean±SEM. (G) Decreased first and second phase insulin secretion in pregnant ß-ARNT versus floxed control females. Insulin expressed as percentage of baseline. *p<0.05. Data points represent mean±SEM.

Before pregnancy, β-ARNT dams had normal fasting glucose and mildly increased peak glucose during glucose tolerance testing (GTT) **(** **Figure 2A** **)**, consistent with our previous report [18]. During pregnancy, β-ARNT mice maintained fasting glucose at a similar level to FCs **(** **Figure 2B** **)** but peak glucose was markedly higher than either pre-pregnancy or in pregnant FCs (p<0.01 for both). Glucose tolerance deteriorated to a much smaller extent in during pregnancy in FC mice, achieving statistical significance only at 30 minutes (p<0.05).

**Figure 2C** shows G16.5 glucose tolerance curves for each individual mouse. Clear separation between the groups is present. β-ARNT dams had no differences in circulating fasting triglyceride levels (**Figure 2D** **).** There was no correlation between maternal glucose tolerance and triglycerides.

### Glucose-stimulated Insulin Secretion (GSIS)

As previously reported, β-ARNT females had impaired GSIS in the non -pregnant state compared to floxed control females, with reduced first phase insulin secretion (p = 0.013) **(** **Figure 2E** **)**. At G16.5, first phase insulin secretion was detectable in both genotypes. However, insulin secretion was markedly impaired in β-ARNT dams, with reduced first and second phase insulin response, whether expressed as raw insulin values **(** **Figure 2F** **)** or percentages of baseline **(** **Figure 2G** **)**.

### Fetal and Newborn Phenotype

**Figure 3:**
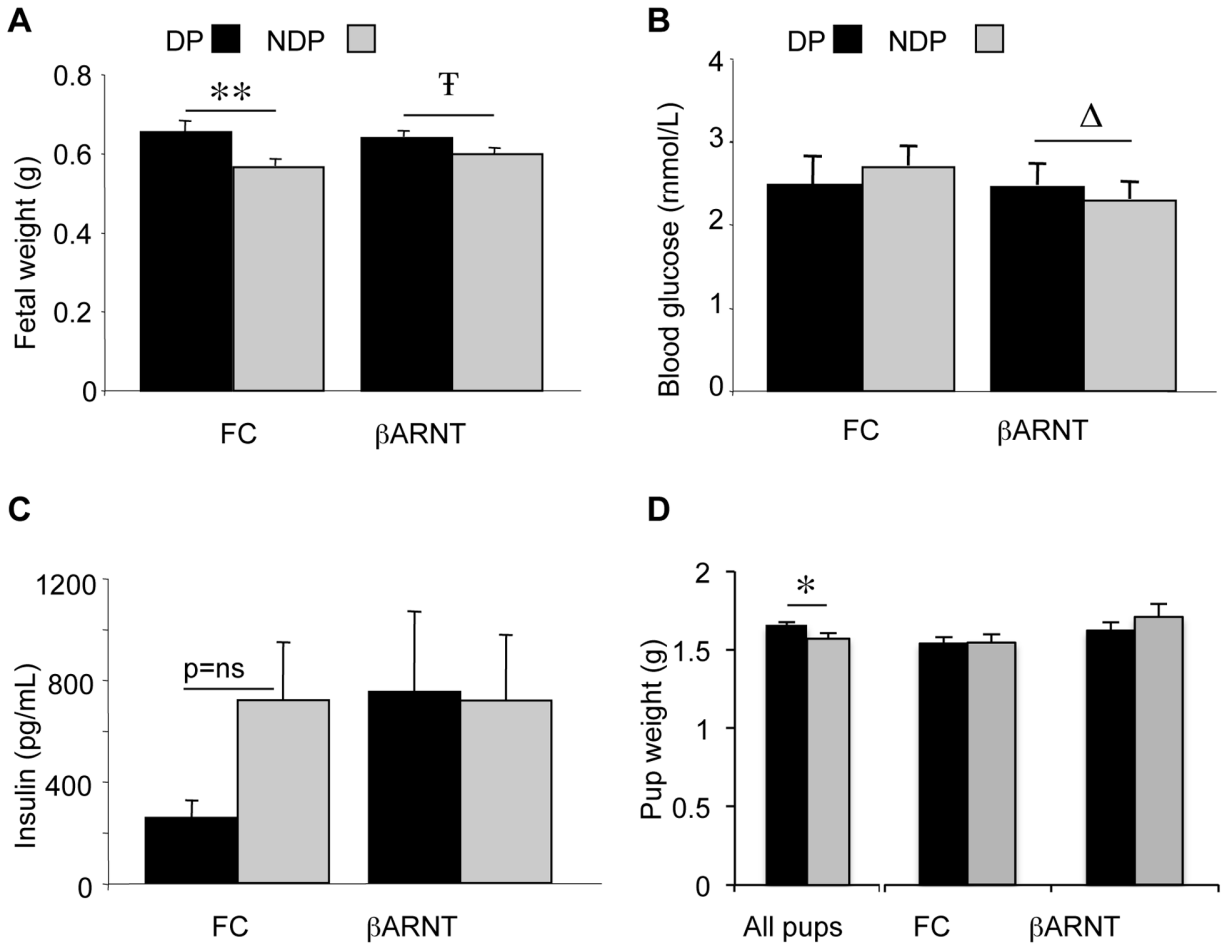
Fetal and newborn phenotype. (A) Weight of floxed control (FC) and ß-ARNT fetuses from diabetic pregnancies (DP) (n = 27 ß-ARNT and 14 FC) and non diabetic pregnancies (NDP) (n = 28 ß-ARNT and 32 FC). **p<0.01, Ŧ = 0.07, t-test. Significant main effect of DP on offspring weight on 2-way ANOVA, p = 0.001. Bars represent mean±SEM. (B) Blood glucose levels in floxed control and ß-ARNT fetuses from diabetic pregnancies and non diabetic pregnancies. Δ = 0.12, t-test. Bars represent mean±SEM. (C) Plasma insulin levels in floxed control and ß-ARNT fetuses from diabetic pregnancies (n = 25 ß-ARNT and 13 FC) and non diabetic pregnancies (n = 14 ß-ARNT and 18 FC). Bars represent mean±SEM. (D) Weight of floxed control and ß-ARNT pups from diabetic pregnancies (for all pups: n = 74 from DP and 41 from NDP, for pups in which genotype was known: n = 19 FC pups from DP, 9 from NDP, n = 9 ß-ARNT pups from DP and 10 from DP). *p<0.05, t-test. Bars represent mean±SEM. For this figure, black bars represent diabetic pregnancies and grey bars represent non diabetic pregnancies.

β-ARNT and FC females were mated with the opposite genotype male so that litters comprised of ∼50% β-ARNT and ∼50% FC offspring (**Figure 1**). Gestational day 16.5 fetuses from β-ARNT mothers were heavier than those from FC mothers **(** **Figure 3A** **)**. There was no significant interaction between maternal genotype and pup genotype, but a significant main effect of maternal genotype on fetal weight (p = 0.001, 2-way ANOVA), with fetuses from β-ARNT pregnancies being 0.07±0.02 g heavier than fetuses from FC pregnancies. Blood glucose levels in FC fetuses did not differ significantly depending on the maternal genotype. In β-ARNT fetuses, there was a trend to higher blood glucose levels in those from β-ARNT pregnancies than those from FC mothers **(** **Figure 3B** **)**. However, 2-way ANOVA analysis of blood glucose levels showed there was no significant interaction between maternal genotype and pup genotype, and no significant main effects of maternal genotype or pup genotype. Two-way ANOVA analysis of fetal insulin levels showed there was no significant interaction between maternal genotype and pup genotype, and no significant main effects of maternal genotype or pup genotype on fetal insulin **(** **Figure 3C** **)**.

Pups were weighed at 1.5 days of age. Analysis of pup weight was restricted to litters which had 6–8 pups because newborn weights are well described to correlate inversely with litter size [24]. Within these litters, in which pups were not separated by genotype or sex, pup weights were greater in the offspring of β-ARNT versus floxed-control pregnancies (1.66±0.02 g versus 1.57±0.03 g, p = 0.02, t-test). Not all pups were genotyped at this age as some were used for studies at later time-points. In the smaller group of pups which were culled and thus in which genotype was known, there was no difference in weight in pups from diabetic versus non diabetic pregnancies when separated by genotype, with 9–19 pups per group. Two-way ANOVA showed no significant interaction between maternal genotype and pup genotype, and no significant main effect of maternal genotype **(** **Figure 3D** **)**.

### Beta-cell Mass and Morphology

**Figure 4:**
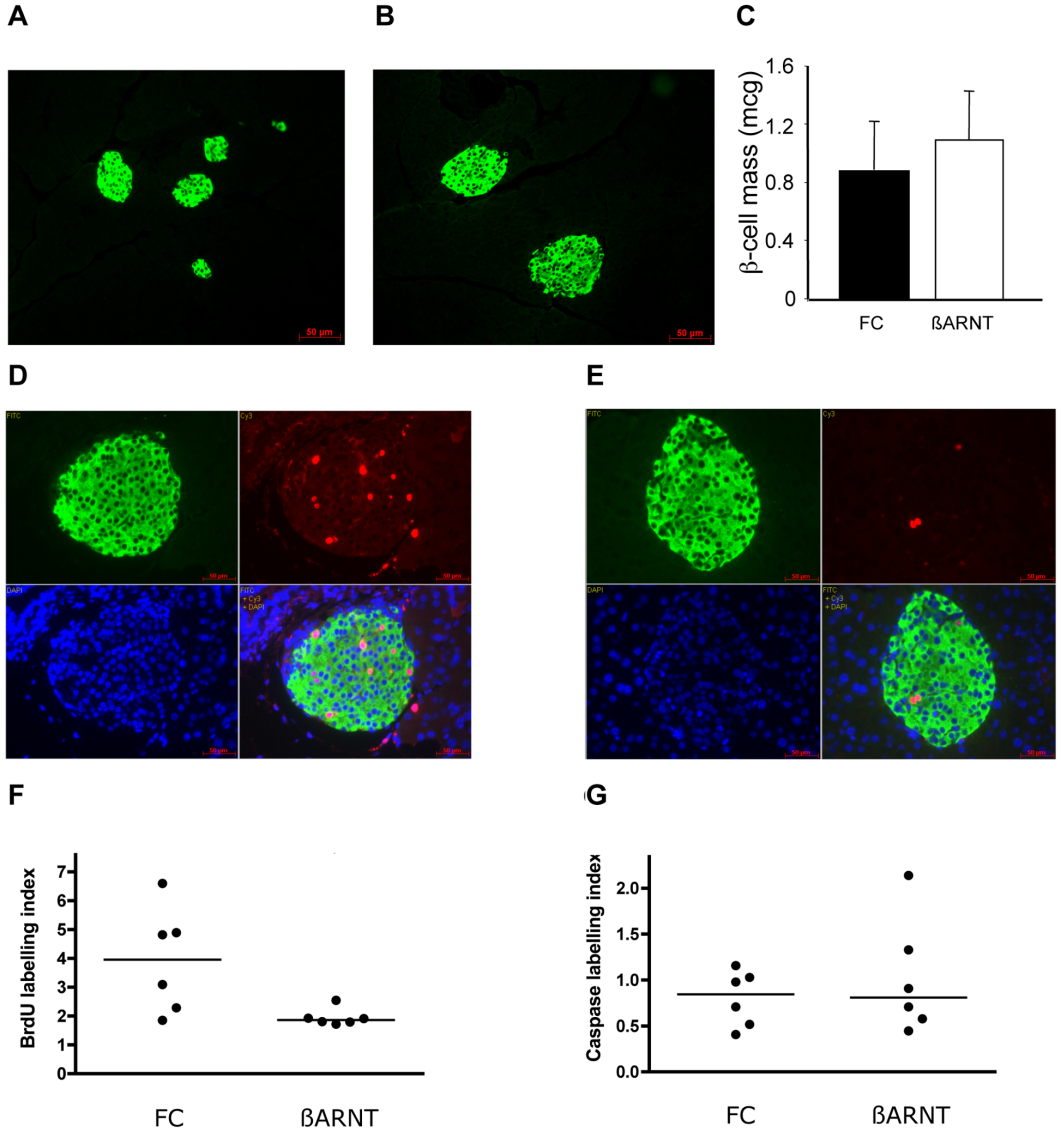
Islet histology in ß-ARNT and floxed control mothers. (A–B) Representative images of insulin immunofluorescence on islets from a floxed control (A) and ß-ARNT (B) mouse in late pregnancy. (C) Beta-cell mass in floxed control (FC) (n = 6) and ß-ARNT (n = 6) females in late pregnancy. p = ns, t-test. (D–E) Representative BrdU (red), insulin (green) and DAPI (blue) immunofluorescence on islets from a (D) floxed control and (E) ß-ARNT mouse in late pregnancy. (F) The BrdU labelling index was significantly decreased in insulin-positive islet cells of ß-ARNT versus floxed control (FC) dams (median 1.86 (1.72–2.55) versus 3.96 (1.86–6.6)) *p<0.05, Mann Whitney test). Data points represent individual mice. (G) The caspase labelling index was similar in insulin-positive islet cells of ß-ARNT versus floxed control dams (median 1.69 (0.48–1.26) versus 0.84 (0.56–3.01) p = NS, Mann Whitney test). Data points represent individual mice.

A separate cohort of floxed-control and β-ARNT females was sacrificed at 15.5 days of gestation for assessment of islet mass and beta-cell proliferation (Cohort 3, Figure 1). This time-point is consistent with the reported peak in beta-cell proliferation in murine pregnancy [25]. Representative images of floxed-control and β-ARNT islets are shown in **Figures 4A and 4B**. Quantitation confirmed that β-ARNT and floxed-control dams had similar mean calculated beta-cell mass (p>0.7, **Figure 4C** **)**, beta-cell area (4001±511 versus 4024±505 µm^2^, p = 0.98) and proportional beta-cell area (percentage of total sectional area) (0.30±0.07 versus 0.31±0.04%, p = 0.98). Although β-ARNT dams had significantly worse glucose tolerance, beta-cell mass was similar at that time of gestation. Since the β-ARNT dams had worse glucose tolerance, the similar beta-cell mass reflects inappropriate compensation.

### Beta-cell Proliferation and Apoptosis in Pregnancy

BrdU/insulin immunofluorescence demonstrated a significant proportion of beta-cells were BrdU positive in pregnant floxed-control dams, as expected (**Figure 4D**). There was obviously decreased BrdU incorporation in β-ARNT dams **(** **Figure 4E** **)**. Quantitation confirmed a 50% lower BrdU staining index in β-ARNT versus floxed-control dams at day 15.5 of gestation (p = 0.026, **Figure 4F** **)**. These results demonstrate that β-ARNT females have a reduced capacity for beta-cell proliferation in the setting of impaired glucose tolerance in pregnancy. This indicates failure to increase beta-cell mass to compensate for their increased glucose levels.

Pregnant floxed-controls showed BrdU staining in pregnancy that was clearly higher than non-pregnant females. Beta-cell apoptosis during pregnancy was not different in the two groups; cleaved caspase-3 staining in beta-cells was similar in β-ARNT and FC dams at day 15.5 of gestation. The caspase staining index is shown in (**Figure 4G** **)**.

### Islet Gene Expression in Pregnancy

**Figure 5:**
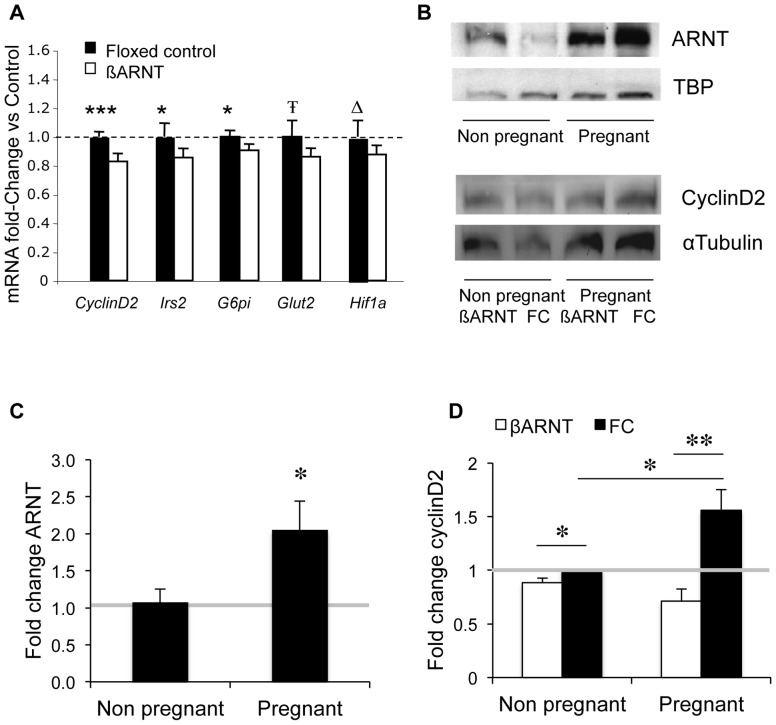
Gene and protein expression: ARNT and cyclinD2. (A) Gene expression in β-ARNT islets (white bars) expressed as percentage of gene expression in floxed control islets (black bars) (n = 13 β-ARNT and 13 FC). ***p<0.001, **p<0.01, *p<0.05, Ŧ p = 0.06, Δ p = 0.08, t-test. Bars represent mean±SEM. (B) Western blot of ARNT in islets from non pregnant and pregnant wild type females (top) Western blot of cyclinD2 in islets from non pregnant and pregnant floxed control and β-ARNT females (bottom). (C) Quantitation of ARNT protein in wild type female islets, expressed as fold change of non pregnant islets (n = 6 vs. 6). *p = 0.048, t-test. Bars represent mean±SEM. (D) Quantitation of cyclinD2 protein in non pregnant and pregnant β-ARNT (white bars) and floxed control (black bars) islets, expressed as fold change of non pregnant floxed control islets (n = 4 vs. 4). *p = 0.02, **p = 0.002, t-test. Bars represent mean±SEM.

To investigate causes of the difference in rate of beta-cell proliferation, expression of cell cycle genes was measured. There was a 17% decrease in *cyclinD2* expression (p<0.001) in β-ARNT versus floxed control islets. There were also significant reductions in expression of *Irs2* and *G6PI*, and trends to reduced *Glut2* and *Hif1a* expression **(** **Figure 5A** **)**. There were no differences in expression of *Ccne1, Ccnd3, Bcl2, Cdk2, Cdk4, Hnf1a, Stat3, Stat5a, Prlr, Akt2* or *Glut1* (data not shown).

### Islet CyclinD2 and ARNT Protein Increase in Normal Pregnancy

ARNT protein was also assessed in pregnant and non pregnant islets from normal females. There was a 2.1±0.4 fold increase in ARNT during late gestation (p = 0.048, **Figure 5B and 5C**).

Because cyclinD2 mRNA was decreased in β-ARNT islets, we assessed cyclinD2 protein in islets of non pregnant and pregnant β-ARNT and floxed control females by Western blot. Non pregnant β-ARNT and floxed control females had similar levels of cyclinD2. In late pregnancy, cyclinD2 increased 1.6±0.2 fold in floxed controls (p<0.05), but not in β-ARNT females (p = ns). Thus β-ARNT mice had decreased cyclinD2 protein in pregnancy (0.7±0.1 fold versus non-pregnant controls, p = 0.002). A representative Western blot is shown in **Figure 5B**, and quantitation is shown in **Figure 5D**. Because ARNT is a transcription factor, chromatin immunoprecipitation assays (ChIP) were performed and the proximal cyclinD2 promoter was amplified by PCR to determine whether regulation was direct. There was no enhancement with ARNT immunoprecipitation compared to the input fraction, suggesting that the regulation of Cyclin D2 is probably indirect.

## Discussion

GDM occurs when pancreatic beta-cells are unable secrete sufficient insulin to keep up with the increasing demands of pregnancy [6], [7]. The normal insulin response is a result of a left-shift in the glucose-response curve and increased beta-cell mass [4], [5]. We have previously reported that the transcription factor *ARNT* is reduced in islets of humans with type 2 diabetes [18]. Using a mouse model with a beta-cell specific deletion of *Arnt*, we now demonstrate that *Arnt* may also play a critical role in upregulating beta-cell function and proliferation during normal pregnancy.

In the non-gravid state, the β-ARNT mouse demonstrates that ARNT did not appear to be required for fetal beta-cell development but is required for normal adult beta-cell function. Defective first phase insulin secretion in adult mice results in mild glucose intolerance compared to floxed controls. This is associated with decreased expression of a number of genes that regulate glucose sensing, glycolysis and insulin secretion. Similarly, in human T2D islets, reduced *ARNT* expression is associated with a reduction in expression of glucose metabolic genes including *IRS2, IR, AKT2, G6PI, PFK, PGM* and the maturity onset diabetes of the young (MODY) gene *HNF-4*α [18]. Therefore *ARNT* is a potential master regulator of human and murine ß-cell function in the non-gravid state.

We now show that *ARNT* may also play an important role in the adaptation to the beta-cell stress of pregnancy, either by increasing intrinsic function or proliferation. β-ARNT mice had substantially impaired glucose tolerance in late pregnancy, with a glucose intolerance profile similar to that seen in humans with GDM. The increase in glucose was significant enough to result in increased fetal weight. Pups from β-ARNT mothers were heavier than those from floxed-control pregnancies, whether the pups were control genotype, or β-ARNT. In β-ARNT genotype offspring, there was also a trend to increased fetal blood glucose levels in those from β-ARNT pregnancies. This picture is similar to human GDM, which is associated with increased fetal growth and exposure to higher glucose levels.

Glucose-stimulated insulin secretion in β-ARNT dams confirmed that their impaired glucose tolerance was due to a beta-cell defect. β-ARNT islets demonstrated decreased BrdU staining but similar caspase staining, suggesting that beta-cell proliferation was decreased without increase in apoptosis during pregnancy. β-ARNT females were not able to compensate for the underlying beta-cell defect by appropriately increasing beta-cell mass through proliferation or hypertrophy.

In normal pregnancy, ARNT protein increased in islets of normal females in the last trimester of pregnancy. We have previously reported that islet ARNT expression is not altered in models of insulin resistance including the ob/ob and db/db mouse [18]. ARNT was, however, increased in islets in pregnancy, and this suggests the possibility that ARNT is regulated by lactogenic hormones.

CyclinD2 protein levels also increased in islets of floxed control mice during pregnancy, but not in β-ARNT mice. Thus in pregnancy, both cyclinD2 mRNA and protein levels were increased in floxed control mice compared to β-ARNT mice. These results suggest that the cell cycle regulator gene cyclinD2, an essential regulator of postnatal beta-cell growth, plays a role in promoting ß-cell proliferation in pregnancy, and that it requires ARNT to achieve optimal levels.

As well as impaired beta-cell proliferation, β-ARNT mice had a tendency to lower *Glut2* mRNA and had lower *G6pi* expression, both of which would contribute to impaired glucose sensing. Thus a loss of *Arnt* may result in impaired intrinsic beta-cell insulin secretion as well as a decreased capacity for proliferation.

In the non pregnant state, β-ARNT females only had a mild impairment of glucose intolerance, consistent with our previous report [18]. One explanation could be that ARNT is not essential for ß-cell development, but is required for optimal ß-cell compensation in the face of stressors such as pregnancy.

This model suggests that ARNT, a transcription factor decreased in type 2 diabetic human islets, is upregulated in normal pregnancy, regulates cyclinD2 and is required for the normal augmentation of insulin secretion and beta-cell proliferation that occurs in pregnancy in order to maintain normal glucose tolerance.
